# A quality assurance for respiratory gated proton irradiation with range modulation wheel

**DOI:** 10.1002/acm2.12526

**Published:** 2018-12-30

**Authors:** Keisuke Yasui, Akira Shimomura, Toshiyuki Toshito, Kenichiro Tanaka, Kumiko Ueki, Rie Muramatsu, Masaki Katsurada, Naoki Hayashi, Hiroyuki Ogino

**Affiliations:** ^1^ Faculty of Radiological Technology School of Health Sciences Fujita Health University Toyoake Aichi Japan; ^2^ Nagoya Proton Therapy Center Nagoya City West Medical Center Nagoya Aichi Japan

**Keywords:** proton therapy, quality assurance, range modulation wheel, respiratory gating

## Abstract

The purpose of this study was to provide periodic quality assurance (QA) methods for respiratory‐gated proton beam with a range modulation wheel (RMW) and to clarify the characteristics and long‐term stability of the respiratory‐gated proton beam. A two‐dimensional detector array and a solid water phantom were used to measure absolute dose, spread‐out Bragg peak (SOBP) width and proton range for monthly QA. SOBP width and proton range were measured using an oblique incidence beam to the lateral side of a solid water phantom and compared between with and without a gating proton beam. To measure the delay time of beam‐on/off for annual QA, we collected the beam‐on/off signals and the dose monitor‐detected pulse. We analyzed the results of monthly QA over a 15‐month period and investigated the delay time by machine signal analysis. The dose deviations at proximal, SOBP center and distal points were −0.083 ± 0.25%, 0.026 ± 0.20%, and −0.083 ± 0.35%, respectively. The maximum dose deviation between with and without respiratory gating was −0.95% at the distal point and other deviations were within ±0.5%. Proximal and SOBP center doses showed the same trend over a 15‐month period. Delay times of beam‐on/off for 200 MeV/SOBP 16 cm were 140.5 ± 0.8 ms and 22.3 ± 13.0 ms, respectively. Delay times for 160 MeV/SOBP 10 cm were 167.5 ± 15.1 ms and 19.1 ± 9.8 ms. Our beam delivery system with the RMW showed sufficient stability for respiratory‐gated proton therapy and the system did not show dependency on the energy and the respiratory wave form. The delay times of beam‐on/off were within expectations. The proposed QA methods will be useful for managing the quality of respiratory‐gated proton beams and other beam delivery systems.

## 1 INTRODUCTION

In passive proton therapy systems, a range modulation wheel (RMW) is widely used to deliver a uniform depth dose to the target volume. The RMW rotates and is gated with the proton beam output to provide a spread‐out Bragg peak (SOBP).^1^ Proton therapy for moving tumors has been investigated with passive or active scanning methods in many studies.^2^, ^3^, ^4^ In general, the passive proton beam is more robust for moving tumors than active scanning methods. One technique to manage respiratory organ motion is to provide an extra margin in the treatment planning^5^ and to use a respiratory gating system.^6^, ^7^ To avoid dose uncertainties and limitations introduced by incomplete modulations, the passive proton therapy system with RMW features complete modulation cycles.^8^

Quality assurance (QA) for respiratory gating therapy must include patient management and the respiratory gating system in tandem with the irradiation technology. The AAPM TG‐76 report^9^ indicates various configurations and techniques for respiratory gating and recommends that technology‐specific QA should be employed. The AAPM TG‐142 report^10^ provides criteria and tolerances for respiratory‐gated photon beams, and implementation of these tests has been reported.^11^ These reports recommend that beam output and energy consistency be assured every month and year. In proton therapy, energy consistency is very important because the proton beam has a range that corresponds to its energy. Kase et al^12^ reported a QA procedure for a proton therapy system including respiratory gating. However, they mentioned only output consistency with respiratory gating. A report on the proton machine QA procedures of the MD Anderson Cancer Center has also been published,^13^ but did not mention respiratory gating. Since the RMW proton beam is synchronized with beam output, it should be included in QA for the stabilities of beam output, SOBP width and beam range. Furthermore, complete modulation cycles must operate with respiratory gating, which can cause dose deviation or an irregular SOBP shape. In this study, we for the first time provide QA methods and clarify the characteristics and long‐term stability of the respiratory‐gated proton beam with RMW.

## 2 MATERIALS AND METHODS

### 2.A Beam delivery system

**Figure 1:**
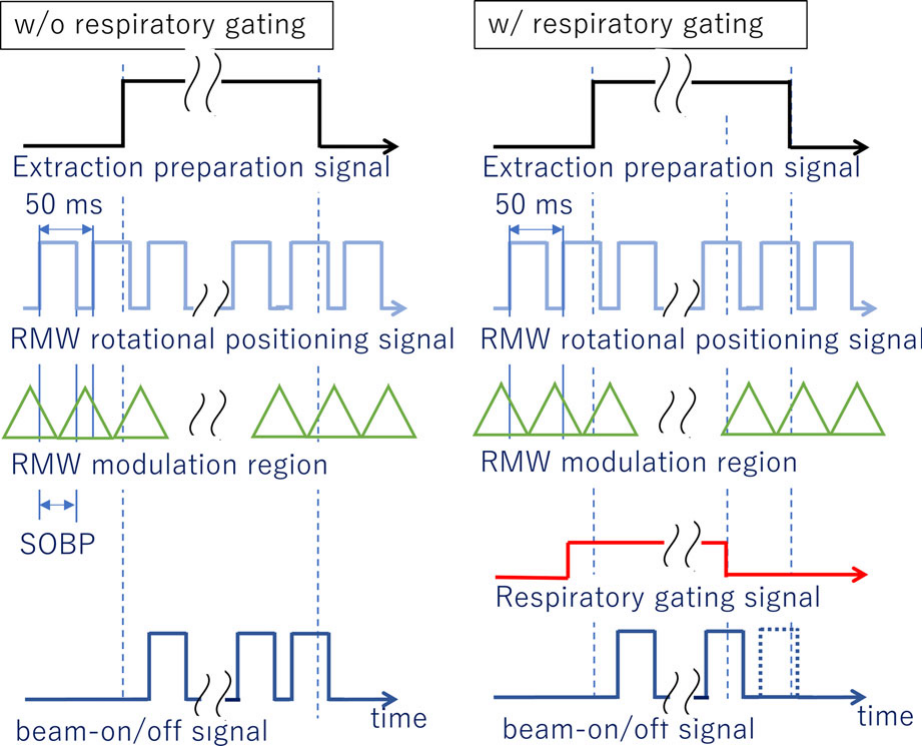
Relationships of all signals to the generation beam‐on/off signal. Left figure showed without (w/o) a respiratory gating pattern and right figure show with (w/) the gating pattern.

The accelerator at the Nagoya Proton Therapy Center (NPTC) is a synchrotron with a linac injector. The NPTC has both the passive scattering system and the spot scanning system. The synchrotron at the NPTC can produce 8 energies (from 100 to 250 MeV) for the passive method and 95 energies (from 71.6 to 221.4 MeV) for the active scanning method. For the active scanning method, the energy of each beam can be changed spill to spill. The maximum spill length for the passive method is 0.5 s and the active scanning method is 4.4 s. The duration of flat top length is variable length and the maximum flat top length is currently set to be 5 s. The acceleration and deceleration time are about 1 s. In the respiratory gating proton therapy, we mainly use the passive scattering system. Characteristics of the passive scattering system at the NPTC comprises mounted RMWs and the MLC system^14^ RMWs of the NPTC rotates at 400 rpm and have six modulation regions per rotation. The RMW has an encoder to monitor the angle of the wheel. The RPM rotational positioning signal, that means the signal to make arbitrary SOBP width, is generated across two modulation regions. Therefore, the cycle of the RPM rotational positioning signal is 50 ms as shown in Fig. 1. During treatment, the beams can be tuned on and off synchronized with the predetermined angle of the wheel and SOBP width can be varied with the predetermined angle. The beam‐on signal is generated from two input signals: the extraction preparation signal and the RMW rotational position signal. The extraction preparation signal is turned on after the accelerator reached the flat top and turned off when the accelerator ready for deceleration. In respiratory‐gated proton therapy, a respiratory gating signal is added to generate the beam‐on signal. Figure 1 shows the relationship of each signal to extract proton beam. The beam‐on/off signal is generated when the all related signals are assembled, however, to generate the specified SOBP width completely, beams are not turned on or off when the RMW rotational position signal is on or off (complete modulation cycles^8^). Beam output have a delay time from the respiratory‐gated signal due to the signal transmission and complete modulation cycles. Since we use respiratory gating with free breathing, the delay time of our RMW system with respiratory gating is at random. When the predetermined MU is delivered upon expiration, the beam is turned off immediately.

### 2.B Experimental materials and setup for the monthly QA

**Figure 2:**
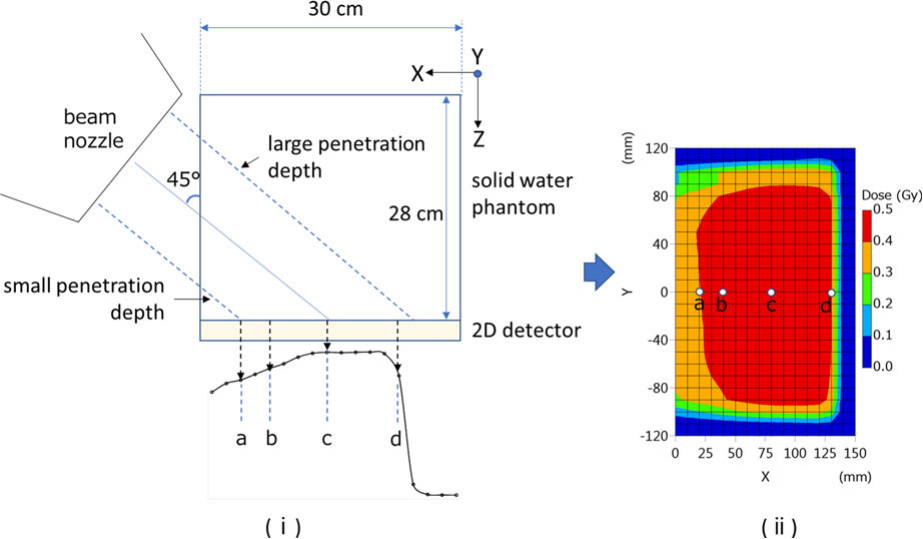
(i) Left figure shows the experimental setup of the SOBP for dose measurements w/and w/o the respiratory gating. Symbols a–d in the SOBP indicate proximal (a,b), SOBP center (c), and distal (d) points. (ii) Right figure shows the example of 2D‐dose distribution obtained by the setup of (i) and symbols a–d.

All measurements and analysis were performed using a general‐purpose solid water phantom (tough water phantom, Kyoto Kagaku), a two‐dimensional ionization chamber array (2D‐ARRAY 729, PTW) and dose analysis software (Verisoft, PTW). We used a respiratory monitoring system (AZ‐733V, Anzai Medical) to generate a simulated respiratory wave and a respiratory gating signal. This respiratory monitoring system has a 52.8 ms time delay from input to output of the respiratory signal. In this study, we used simulated waves that do not have this 52.8‐ms time delay. The simulated wave had a 3‐s sine cycle and the gating‐on period was set at 0.75 s. As mentioned above, to verify stabilities of SOBP width and beam range is important because of the proton beam has range. Furthermore, the RMW proton beam system is synchronized with the beam‐on/off signal that also related to the respiratory gate‐on/off signal in the respiratory‐gated proton therapy. To measure the SOBP width and beam range with respiratory gating, we devised an easily method using an oblique incidence beam to the lateral side of a solid water phantom and measured the 2D‐dose distribution with the two‐dimensional detector. The size of a solid water phantom is 30 cm square and we stack a solid water phantom up to 28 cm as shown in Fig. 2. The purpose of monthly QA is to verify the change from baseline data and this easily method is enough for monthly QA. By using oblique incidence beam, proton beam of off‐center axis through large penetration depth and that showed beam range. Another side of off‐center axis beam through small penetration depth and that showed proximal region. This method can obtain SOBP easily and can compare output, SOBP width and beam range between respiratory gating on and off. The measurement setup, the example of measurement SOBP and 2D‐dose distribution were shown in Fig. 2. We analyzed four points doses that were two proximal (a, b), SOBP center (c), and distal (d) points, as indicated in Fig. 2, because respiratory gating may cause deviation of output, energy and SOBP width due to the error of complete modulation cycles or other signal transmission errors. Measurement doses with (w/) gating were compared with without (w/o) gating. The periodic monthly QA was performed with the 200 MeV/SOBP 10 cm, which was the reference condition at the NPTC.

### 2.C Delay time measurements for the annual QA

**Figure 3:**
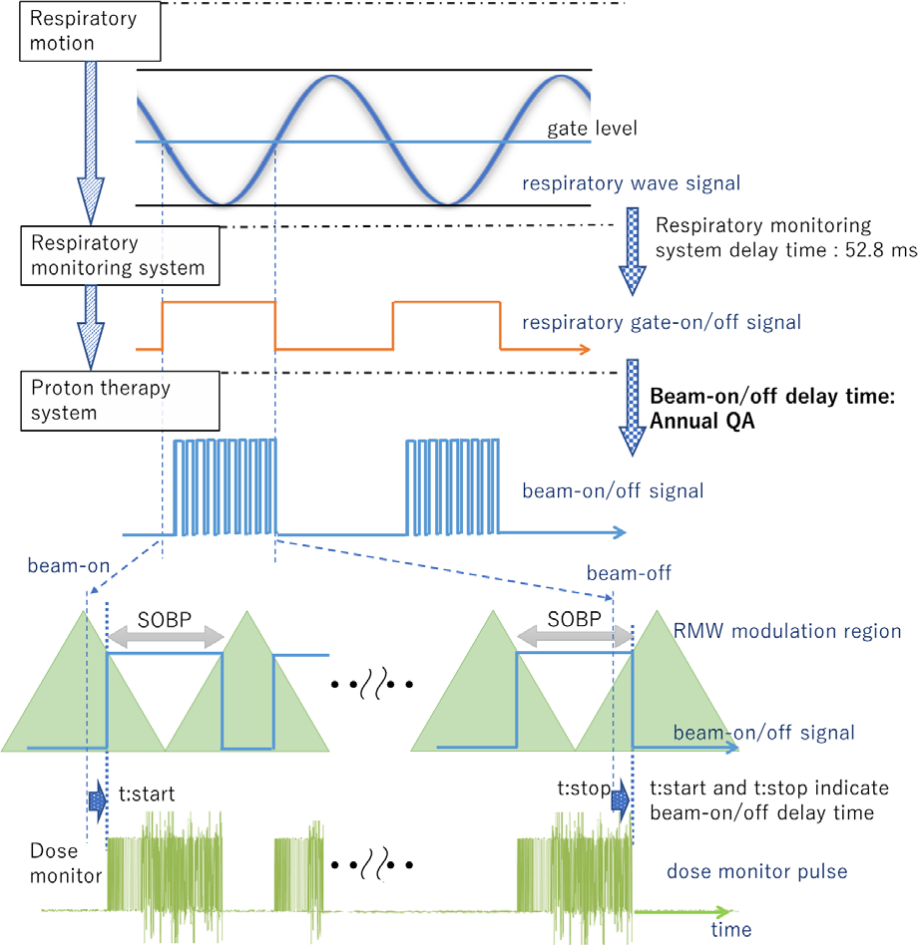
Flowchart of the respiratory motion signal and relationships of the respiratory wave signal, gate‐on/off signals, beam‐on/off signals, RMW modulation regions, and the dose monitor pulse. *t*:start and *t*:stop in figure showed beam‐on/off delay times due to complete modulation cycles and machine delay time.

As the annual QA, we observed three signals: the simulated respiratory wave signal, the respiratory gate‐on/off signal, and the dose monitor pulse. The relationships of respiratory wave signal, gate‐on/off signal, beam‐on/off signal, RMW modulation region, and dose monitor pulse are shown in Fig. 3. The respiratory wave signal for the annual QA is simulated patient wave generated by the respiratory monitoring system (the first row of Fig. 3). The Respiratory gate‐on/off signal, showed in the second row of Fig. 3, was generated by the respiratory gating system and included a 52.8‐ms respiratory monitoring delay time. The third row of Fig. 3 shows beam‐on/off signal mentioned in Fig. 1. The beam‐on/off signals have systematic and random delay relationships with the respiratory gate‐on/off signals of the transmission time and complete modulation cycles. These delay times were shown in the fourth row of Fig. 3. The SOBP shown in Fig. 3 was required RMW modulation region to generate arbitrary SOBP width. The fifth row of Fig. 3 is the dose monitor pulse obtained by the reference dose monitor. Since the reference dose monitor is the nearest to the isocenter plane in all beam monitors, we measured the delay time using the dose monitor pulse of the reference dose monitor. Because there is possibility of the delay times being affected by beam parameters such as SOBP width and energy, we employed two parameters: 200 MeV/SOBP 16 cm and 160 MeV/SOBP 10 cm. The 16‐cm SOBP is the maximum SOBP width at the NPTC. We measured the beam‐on/off delay time ten times for annual QA and summarized the average time and worst case (maximum) delay time. In this study, we measured delay time from respiratory gate‐on/off signal to beam‐on/off signal that is beam‐on/off delay time, as shown in the third‐fifth low in Fig. 3. These delay times do not include the 52.8‐ms respiratory monitoring system delay time.

### 2.D Extra validation: the energy dependence and the effects of the realistic respiratory wave

**Figure 4:**
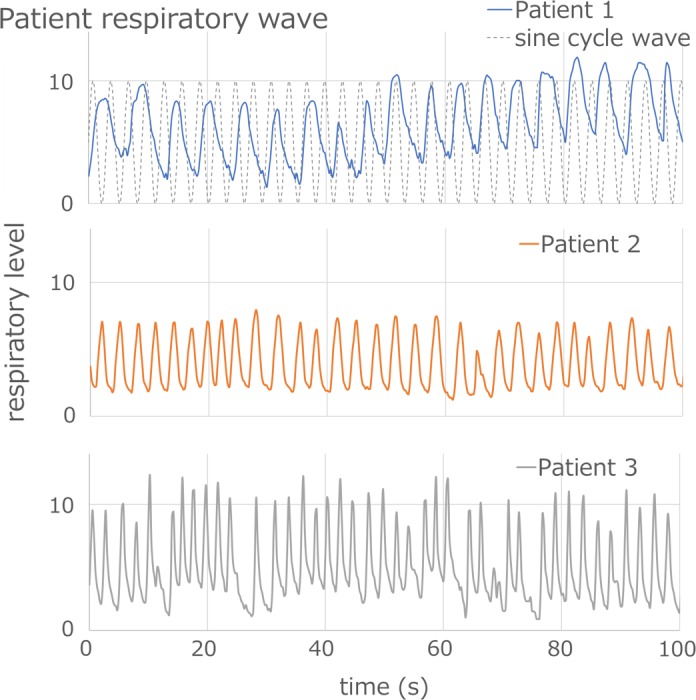
Respiratory waves of three patients that were treated using respiratory gating proton beam at the NPTC. The dashed line in the upper figure is a 3‐s sine cycle curve used in periodic QA.

As the extra validation, we measured the energy dependence to the dose distribution and the effects of the realistic respiratory wave to the dose distribution and delay times. Experimental materials and setup are the same as the monthly QA and the annual QA mentioned at Sections 2.B and 2.C. The energy dependence to the dose distribution was measured using the middle energy (160 MeV/SOBP 10 cm) and the low energy (120 MeV/SOBP 4 cm) proton beam. For the validation of the effects of the realistic respiratory wave, we used real recorded respiratory pattern from three patients that were treated using respiratory proton beam at the NPTC. Respiratory wave forms of these patients are shown in Fig. 4. To measure the effects of the realistic respiratory wave, we used the reference condition (200 MeV/SOBP 10 cm). We measured the energy dependence and the effects of the realistic respiratory wave five times and summarized the average value and worst case.

## 3 RESULTS

**Figure 5:**
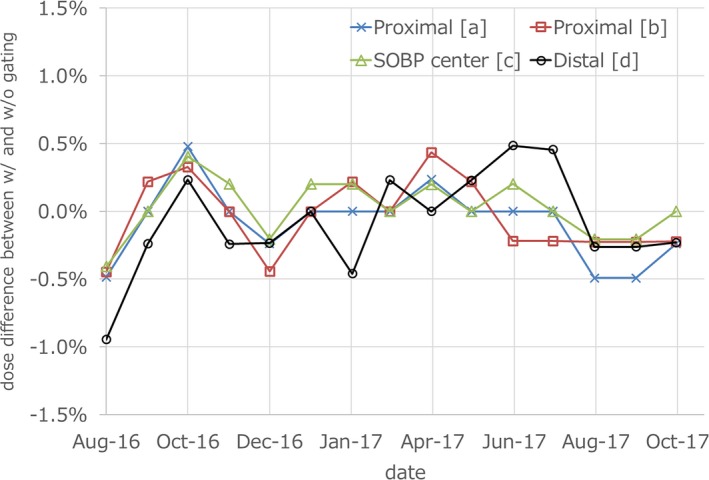
Periodic QA results over a 15‐month period. Symbols a–d correspond to points indicated in Figs. 2 and 6.

Figure 5 shows monthly QA results over a 15‐month period. The dose deviations at proximal, SOBP center and distal were −0.083 ± 0.25%, 0.026 ± 0.20%, and −0.083 ± 0.35%, respectively. The maximum dose deviation between w/and w/o gating was −0.95% at distal and other deviations were within ±0.5%.

**Figure 6:**
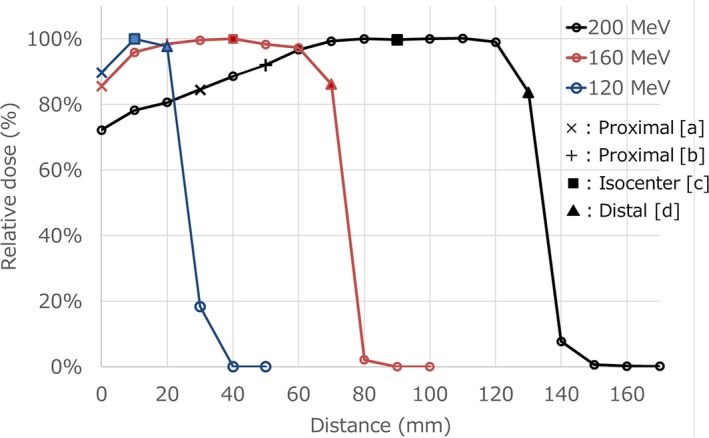
Relative dose curves as a function of the distance from edge of detector for three energies and measurement points used to compare the dose between w/and w/o gating.

Figure 6 shows relative dose curves as a function of the distance from edge of detector for three energies and measurement points used to compare the dose between w/and w/o gating. The low energy beam has only three measurement points, therefore we evaluate three points doses. Table 1 shows dose differences between w/and w/o gating of each measurement point with three energies and three realistic respiratory waves. From these results, there are no energy dependence and the effects of the respiratory wave.

**Table 1:** Dose differences between w/and w/o gating of each measurement point with three energies and three random respiratory waves. The data of the reference condition (200 MeV/SOBP 16 cm) is the average value of over a 15‐month period. Others are average values of five times measurements. Each measurement point is described in Fig. 6

(%)	Measurement point	Average difference	SD	Maximum difference
Reference setting 200 MeV/SOBP 10 cm monthly QA	Proximal [a]	−0.08	0.25	−0.49
	Proximal [b]	−0.04	0.26	−0.45
	Isocenter [c]	0.03	0.20	−0.41
	Distal [d]	−0.08	0.35	−0.95
160 MeV/SOBP 10 cm	Proximal [a]	0.57	0.15	0.63
	Proximal [b]	0.55	0.15	0.69
	Isocenter [c]	0.40	0.12	0.63
	Distal [d]	1.02	0.20	1.18
120 MeV/SOBP 4 cm	Proximal [a]	0.11	0.17	0.54
	Isocenter [c]	−0.40	0.16	−0.48
	Distal [d]	−0.43	0.20	−0.44
Patient wave 1 200 MeV/SOBP 10 cm	Proximal [a]	0.05	0.18	0.24
	Proximal [b]	−0.13	0.11	−0.22
	Isocenter [c]	−0.16	0.20	−0.41
	Distal [d]	0.14	0.31	0.68
Patient wave 2 200 MeV/SOBP 10 cm	Proximal [a]	−0.10	0.12	−0.24
	Proximal [b]	−0.09	0.23	−0.44
	Isocenter [c]	−0.04	0.15	0.20
	Distal [d]	−0.09	0.30	0.45
Patient wave 3 200 MeV/SOBP 10 cm	Proximal [a]	−0.05	0.18	−0.24
	Proximal [b]	−0.09	0.11	−0.22
	Isocenter [c]	−0.24	0.24	−0.61
	Distal [d]	−0.36	0.18	0.45

**Figure 7:**
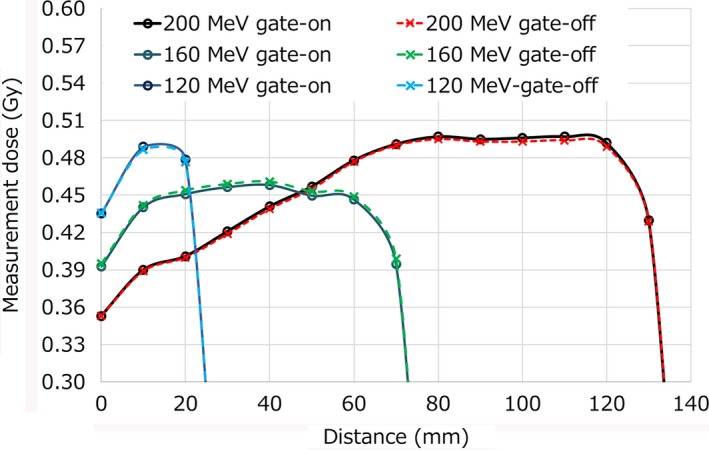
Examples of absolute dose deviations between gate‐on and gate‐off for three energies.

Figure 7 shows an example having maximum absolute dose deviation at the SOBP center. From the result of Fig. 7, SOBPs w/and w/o gating had the same shape and global dose, with only small differences. The same trend was seen in results of other months. Proximal and SOBP center doses showed the same trend over the 15‐month period, while distal doses showed a different trend in some months.

Table 2 shows the results of annual QA and extra validation for beam‐on/off delay times with two different parameters and three realistic respiratory waves. The beam‐on delay time for SOBP 16 cm was more stable and smaller than that for SOBP 10 cm. In contrast, the beam‐off delay time of SOBP 16 cm was less stable and longer than that for SOBP 10 cm. The maximum beam‐on and beam‐off delay times were 199.2 ms and 36.8 ms, respectively. From the results of delay times for patient respiratory waves, our system did not show the effect of the respiratory wave.

**Table 2:** Beam‐on and beam‐off delay times for two energies and beam‐off delay times for three random respiratory waves

	(ms)	Average delay time	SD	Maximum delay time
200 MeV/SOBP 16 cm	Beam‐on	140.5	0.8	142.0
	Beam‐off	22.3	13.0	36.8
160 MeV/SOBP 10 cm	Beam‐on	167.5	15.1	199.2
	Beam‐off	19.1	9.8	30.4
Patient wave 1 200 MeV/SOBP 10 cm	Beam‐off	23.0	11.3	39.0
Patient wave 2 200 MeV/SOBP 10 cm	Beam‐off	19.2	11.4	36.0
Patient wave 3 200 MeV/SOBP 10 cm	Beam‐off	28.2	10.6	40.0

## 4 DISCUSSION

We reported periodic QA results of the respiratory gated proton beam and beam‐on/off delay times when using RMW. In the TG‐142 report, the tolerance of beam output constancy with the respiratory gating is recommended to be within 2%.^10^ With regard to the QA of respiratory gating treatment, proton therapy systems are required to have the same accuracy.^12^ From the results of this report, our system has sufficient stability. From the results of Figs. 6, 7 and Table 1, it is assumed that the dose deviation has no energy dependence and the effects of respiratory waves. As shown in Fig. 7, the SOBP shapes between w/and w/o gating showed no remarkable change during a 15‐month period, indicated RMWs worked well regardless of respiratory gating. Beam energy constancy can be verified by the distal dose because 1‐mm range error cause more than a 5% dose difference, so the range errors of our measurements were estimated to be less than 0.2 mm. The small dose deviations between w/and w/o gating were within the reproducibility of the dose output. Delay times for passive proton‐based gating systems had been reported to be 65–195 ms and a linac‐based gating system had a delay time of 170 ± 30 ms.^8^, ^15^ The results of beam‐on delay times in this study was almost the same as in previous reports. However, our beam‐off delay times were significantly smaller. In addition, the large SOBP had long beam‐off delay time and larger variances. These results showed the need to wait for complete modulation of the RMW. From the results of the beam‐off delay times for patient respiratory waves, our system did not have the dependence of the respiratory wave form. This result shows that the delay time of proton system with RMW depend on only beam‐on/off signal. In the clinical setting, the 52.8‐ms time delay of our respiratory gating system is added to machine time delay, and an extra gating margin is required to manage total delay times. The maximum total delay time of beam‐off of NPTC machine was 36.8 + 52.8 = 89.6 ms. According to the AAPM TG‐142 report, the speed of moving object was no greater than 20 mm/s. From our results, the assumed maximum movement of object in our passively proton therapy system is 1.8 mm. Clinically, we have added extra 3 mm gating margin in the anterior direction to manage the impact of the beam‐off delay time. Previous studies have also reported imaging time delays and energy dependences.^16^, ^17^ Further investigation is required to determine the optimal extra gating margin for the respiratory‐gated proton therapy using RMW.

In this study, we used an oblique incidence beam. An oblique incidence beam can detect the beam range and doses at multiple depths simultaneously with the same dose and the beam intensity of the clinical setting. Ideally, the multi‐layer ionization chamber (MLIC) is useful to detect the beam range and the SOBP width, however, the oblique incidence beam is enough to verify the stabilities of the proton beam.

## 5 CONCLUSION

We proposed periodic QA methods and clarified the stabilities of dose output, SOBP width, beam range and beam‐on/off delay time of respiratory‐gated proton beam with RMW. An oblique incidence beam was useful to manage the quality of respiratory‐gated proton beam and can be applied to other systems, such as scanning technique, to easily measure doses at multiple depths and beam energy constancy. Our beam delivery system with RMW showed enough stability to the respiratory gating therapy. Delay times of beam‐on/off were within expectations in respiratory gated proton therapy. The system is used for treatment of lung, liver, pancreas, and other tumors with respiratory motion.

## CONFLICT OF INTEREST

The authors have no conflicts of interest directly relevant to the content of this article.
